# Reintubation rates after extubation to different non-invasive ventilation modes in preterm infants

**DOI:** 10.1186/s12887-021-02760-7

**Published:** 2021-06-16

**Authors:** Alaa Masry, Nuha A. M. A. Nimeri, Olfa Koobar, Samer Hammoudeh, Prem Chandra, Einas E. Elmalik, Amr M. Khalil, Nasir Mohammed, Nazla A. M. Mahmoud, Lisa J. Langtree, Mohammad A. A. Bayoumi

**Affiliations:** 1 Neonatal Intensive Care Unit (NICU), Women’s Wellness and Research Center (WWRC), Hamad Medical Corporation (HMC), P.O. Box 3050, Doha, Qatar; 2 Medical Research Center, Hamad Medical Corporation (HMC), P.O. Box 3050, Doha, Qatar; 3 Medical Records Department, Women’s Wellness and Research Center (WWRC), Hamad Medical Corporation (HMC), P.O. Box 3050, Doha, Qatar

**Keywords:** Nasal intermittent positive pressure ventilation, Nasal continuous positive airway pressure, Respiratory distress syndrome, Reintubation, Newborn

## Abstract

**Introduction:**

Respiratory Distress Syndrome (RDS) is a common cause of neonatal morbidity and mortality in premature newborns. In this study, we aim to compare the reintubation rate in preterm babies with RDS who were extubated to Nasal Continuous Positive Airway Pressure (NCPAP) versus those extubated to Nasal Intermittent Positive Pressure Ventilation (NIPPV).

**Methods:**

This is a retrospective study conducted in the Neonatal Intensive Care Unit (NICU) of Women’s Wellness and Research Center (WWRC), Doha, Qatar. The medical files (*n* = 220) of ventilated preterm infants with gestational age ranging between 28 weeks 0 days and 36 weeks + 6 days gestation and extubated to non-invasive respiratory support (whether NCPAP, NIPPV, or Nasal Cannula) during the period from January 2016 to December 2017 were reviewed.

**Results:**

From the study group of 220 babies, *n* = 97 (44%) babies were extubated to CPAP, *n* = 77 (35%) were extubated to NIPPV, and *n* = 46 (21%) babies were extubated to Nasal Cannula (NC). Out of the *n* = 220 babies, 18 (8.2%) were reintubated within 1 week after extubation. 14 of the 18 (77.8%) were reintubated within 48 h of extubation. Eleven babies needed reintubation after being extubated to NCPAP (11.2%) and seven were reintubated after extubation to NIPPV (9.2%), none of those who were extubated to NC required reintubation (*P* = 0.203). The reintubation rate was not affected by extubation to any form of non-invasive ventilation (*P* = 0.625). The mode of ventilation before extubation does not affect the reintubation rate (*P* = 0.877). The presence of PDA and NEC was strongly associated with reintubation which increased by two and four-folds respectively in those morbidities. There is an increased risk of reintubation with babies suffering from NEC and BPD and this was associated with an increased risk of hospital stay with a *P*-value ranging (from 0.02–0.003). Using multivariate logistic regression, NEC the NEC (OR = 5.52, 95% CI 1.26, 24.11, *P* = 0.023) and the vaginal delivery (OR = 0.23, 95% CI 0.07, 0.78, *P* = 0.018) remained significantly associated with reintubation.

**Conclusion:**

Reintubation rates were less with NIPPV when compared with NCPAP, however, this difference was not statistically significant. This study highlights the need for further research studies with a larger number of neonates in different gestational ages birth weight categories. Ascertaining this information will provide valuable data for the factors that contribute to re-intubation rates and influence the decision-making and management of RDS patients in the future.

## Introduction

Respiratory Distress Syndrome (RDS) is a common cause of neonatal morbidity and mortality in premature infants. It is caused by a deficiency of surfactant in a premature lung. In preterm infants at-risk for or with established RDS, Nasal Continuous Positive Airway Pressure (NCPAP) is the initial preferred intervention to prevent alveolar collapse [1–3]. It works through splinting the pharyngeal airway with positive pressure, thereby maintaining lung recruitment and reducing the risk of both upper and lower airway collapse and obstruction. Prolonged mechanical ventilation can be associated with significant morbidity in preterm neonates and the practice of early extubation to non-invasive respiratory support has been the focus since the past decade. Neonatologist taking care of preterm babies do their best to avoid intubating those babies as intubation starts the damaging process of those babies’ lungs. If intubated, many Randomized Controlled Trials (RCTs) and meta-analyses have shown NCPAP to be a useful method of respiratory support after extubation [4, 5].

Furthermore, Nasal Intermittent Positive Pressure Ventilation (NIPPV) is a delivery model of positive pressure ventilation that avoids the trauma of the endotracheal placement tube. It augments and promotes the effectiveness of NCPAP by delivering ventilator breaths via nasal prongs or mask [6]. NIPPV Improves both tidal and minute volumes and reduces the inspiratory effort when compared with NCPAP [7]. In other words, it is considered as a bridge before intubation or after extubation. In some situations, things do not go as we wish, and those non-invasive measures; NCPAP and NIPPV fail to maintain adequate functional residual capacity after extubation and reintubation are required.

The popularity of NIPPV is rising since its comparison to NCPAP has demonstrated a significant decrease in respiratory failure, re-intubation rates, and extubation failure [8, 9]. A multi-center randomized clinical trial on 57 preterm infants receiving early surfactant for RDS, reported that NIPPV in comparison to NCPAP reduced the need and duration of mechanical ventilation via an endotracheal tube, and decreased both clinical and physiological Bronchopulmonary Dysplasia (BPD) [10]. There is a paucity of evidence comparing certain non-invasive modalities when used as post-extubation support. In this research study, we aimed to measure and compare the rate of reintubation in preterm babies extubated to different non-invasive respiratory support modalities whether NCPAP, NIPPV, and Nasal Cannula (NC).

## Patients and methods

This retrospective study was conducted in the Neonatal Intensive Care Unit (NICU) of Women’s Wellness and Research Center (WWRC) in Hamad Medical Corporation (HMC), after obtaining ethical approval from the Medical Research Center (MRC-01-18-321) following the Declaration of Helsinki. WWRC is a large tertiary center in Doha, Qatar, with a delivery rate of over 18,000 per year. The medical files of 220 ventilated preterm infants who were born at the gestational age between 28 weeks 0 days and 36 weeks + 6 days gestation and extubated to non-invasive respiratory support in 2016 and 2017 were reviewed. In our unit, we use the Babylog VN500 Ventilator (Dräger, Inc., Luebeck, Germany) to provide NCPAP and NIPPV for extubated infants. The available nasal interfaces in our institution are nasal masks and RAM cannula. All infants were extubated, following our NICU extubation criteria, if Fraction of Inspired Oxygen (FiO2) < 0.30, for a target oxygen saturation of more than 90%, and if a good consistent respiratory effort was present. After extubation, our babies were supported by adequate non-invasive respiratory support of NCPAP pressure of 5–7 cm H2O, or nasal intermittent positive pressure ventilation (NIPPV) PIP pressure of 18–24 cm H2O, or nasal cannula 2–4 Litres/minute. Infants were reintubated and connected to mechanical ventilation if they required high FiO2 of > 0.35–0.40, had frequent or persistent apnea or had respiratory acidosis with pH < 7.25 and PCO2 > 60 mmHg. We traced the rates of reintubation in babies extubated to NCPAP and those extubated to NIPPV. Our primary outcome is to compare the rate of reintubation in those two groups. We identified the need for reintubation by respiratory acidosis, increased oxygen requirement, or apnea that was frequent or severe, leading to the endotracheal tube being reinstated during the week post-extubation [11]. For infants born at more than 28 weeks gestation, we don’t have a special protocol to guide the clinicians to which mode we should extubate to either NIPPV, NCPAP, or NC. It is per the discretion and clinical judgment of the treating physician. As per our local practice, infants less than 28 weeks gestation are extubated to NIPPV, hence, we did not include them in this comparison study. We also excluded infants with multiple congenital anomalies, infants with neuromuscular disorders, and infants with grade III and IV Intra Ventricular Hemorrhage (IVH).

Our secondary outcomes are to compare the characteristics of the two groups to try to identify predictive factors for reintubation and to compare the rate of complications from non-invasive ventilation in both groups.

We designed a data collection sheet with maternal characteristics including nationality, Gestational Age (GA), mode of delivery, antenatal steroids, maternal chorioamnionitis, Premature Rupture of Membranes (PROM), Group B Streptococcus (GBS) status, and maternal comorbidities including Diabetes Mellitus (DM) and hypertensive disorders. Delivery and neonatal variables were also collected including Birth Weight (BW), sex, 1- and 5-min Apgar scores, need for Positive Pressure Ventilation (PPV)/intubation/resuscitation medication at delivery, surfactant therapy, need for a postnatal steroid, Patent Ductus Arteriosus (PDA), pre-extubation mode of ventilation, and duration of NCPAP/NIPPV.

Other outcome measures included gastrointestinal perforation; Necrotizing Enterocolitis (NEC); pneumothorax, Bronchopulmonary Dysplasia, duration of mechanical ventilation, duration of hospitalization, and mortality. BPD was defined according to the definition of the National Institutes of Health Consensus [12]. NEC was diagnosed as stage 2 or more, according to modified Bell’s staging [13]. This is a retrospective study design and for some parameters, the data values were incomplete due to the unavailability of the information in the patients’ record files.

### Statistical analysis

Descriptive statistics were used to summarize data related to demographic, clinical, laboratory, and other characteristics of the study sample. Continuous variables were analyzed using a t-test or a Mann Whitney U test, where applicable, and presented as mean ± standard deviation. Categorical variables were analyzed using a chi-square test or Fisher exact test, where applicable, and presented as frequencies and percentages. A logistic regression model was used to determine the predictors for reintubation. The results were presented as odds ratio and 95% confidence intervals. A receiver operating characteristic (ROC) curve was calculated to evaluate the predictive accuracy of possible predictors. A two-tailed *p*-value of < 0.05 was considered statistically significant. All statistics were computed using SPSS, version 22 (SPSS Inc., Chicago, IL).

## Results

We had 2335 NICU admissions in 2016, and 2411 in 2017. Of these 4746 NICU admissions, 220 infants were born between 28 weeks 0 days and 36 weeks + 6 days gestation and required mechanical ventilation. Most of the study sample were non-Qatari (58.6%) males (55.9%). The mean ± SD for the birth weight in our study population is 1655.4 ± 547.8 g while for the gestational age is 31.3 ± 2.7 weeks (Table 1).

**Table 1:** Demographics of the study sample

Variables	Mean ± SD [median (IQR)]
**Birth weight (gm)**	**1655.4 ± 547.8 [1500 (1242.5, 2038.8)]**
**Gestational Age (Weeks)**	**31.3 ± 2.7 [31 (29, 33)]**
**Apgar Score at 1 min**	**6.7 ± 2.3 [7 (5, 9)]**
**Apgar Score at 5 min**	**8.5 ± 1.5 [9 (8, 10)]**
**Length of hospital stay (Days)**	**38.2 ± 30.7 [31 (18, 49.7)]**
**Duration of Mechanical Ventilation (Days)**	**2.9 ± 3.2 [2 (1, 4)]**
**Duration of NCPAP (Days)**	**3.5 ± 4.7 [2 (1, 4)]**
**Duration of NIPPV (Days)**	**2.8 ± 4.3 [1 (1, 3)]**
**Days till reintubation**	**2.33 ± 1.88 [2 (1, 2.5)]**

*IQR* Inter-quartile range

Out of the total 220 infants that required mechanical ventilation, 97 were extubated to NCPAP, 77 were extubated to NIPPV, and 26 were extubated to NC. Of those 220 babies, 18 (8.2%; 95% CI 5.2 to 12.6%) were reintubated within 1 week after extubation. In our NICU, we use Pressure-Controlled, Assist-Controlled Ventilation with Volume Guarantee as (PC-AC + VG) the primary mode for mechanical ventilation in infants with RDS. Of those intubated babies, 198 were extubated from PC-AC + VG mode, 5 were extubated from Synchronized Intermittent Mandatory Ventilation (SIMV), and 5 were extubated from High-Frequency Oscillatory Ventilation (HFOV) (Table 2).

**Table 2:** Clinical variables of the study sample

Variables	(***n*** = 220) (%)
**Sex**	
**Male**	123 (55.9)
**Female**	97 (44.1)
**Nationality**	
**Qatari**	91 (41.4)
**Non-Qatari**	129 (58.6)
**Diabetic Mother**	
**No**	173 (79)
**Yes**	46 (21)
**Hypertensive Mother**	
**No**	180 (81.8)
**Yes**	40 (18.2)
**Premature Rupture of Membranes (PROM)**	
**No**	181 (82.3)
**Yes**	39 (17.7)
**Chorioamnionitis**	
**No**	212 (96.4)
**Yes**	8 (3.6)
**Maternal Group B Streptococcus Colonization (GBS) Status**	
**Negative**	200 (91.3)
**Positive**	17 (7.8)
**Unknown**	2 (0.9)
**Maternal Antenatal Steroids**	
**No**	108 (49.3)
**Yes**	111 (50.7)
**Need for Positive Pressure Ventilation (PPV) during resuscitation**	
**No**	92 (42.2)
**Yes**	126 (57.8)
**Need for resuscitation medication.**	
**No**	217 (98.6)
**Yes**	3 (1.4)
**Surfactant Administration**	
**No**	66 (30.3)
**Yes**	152 (69.7)
**Number of Surfactant Doses**	
**1**	99 (65.1)
**2**	46 (30.3)
**3**	7 (4.6)
**Need for intubation during resuscitation.**	
**No**	121 (55.3)
**Yes**	98 (44.7)
**Mode of Delivery**	
**Vaginal delivery**	38 (17.4)
**Caesarian Section**	180 (82.6)
**Pre-extubation Ventilation Mode**	
**Pressure-Controlled, Assist-Controlled Ventilation with Volume Guarantee (PC-AC + VG)**	198 (95.2)
**Synchronized Intermittent Mandatory Ventilation (SIMV)**	5 (2.4)
**High-Frequency Oscillatory Ventilation (HFOV)**	5 (2.4)
**Patent Ductus Arteriosus (PDA)**	
**No**	168 (77.8)
**Yes**	48 (22.2)
**Post-extubation mode**	
**Nasal Continuous Positive Airway Pressure (NCPAP)**	97 (48.6)
**Nasal Intermittent Positive Pressure Ventilation (NIPPV)**	77(38.5)
**Nasal Cannula (NC)**	26 (13)
**Reintubation**	
**No**	202 (91.8)
**Yes**	18 (8.2)
**Necrotizing Enterocolitis (NEC)**	
**No**	201 (92.2)
**Yes**	17 (7.8)
**Intestinal Perforation**	
**No**	21 (97.7)
**Yes**	2.3 (2.3)
**Air Leak**	
**No**	196 (89.9)
**Yes**	22 (10.1)
**Bronchopulmonary Dysplasia (BPD)**	
**No**	201 (92.2)
**Yes**	17 (7.8)
**Bronchopulmonary Dysplasia (BPD) Grade**	
**Mild**	11 (64.7)
**Moderate**	6 (35.3)

This is a retrospective study design and for some parameters, the data values were incomplete due to the unavailability of the information in the patients’ record files, and thus all the percentages values were computed using non-missing values

Among the 18 reintubated cases, 14 (77. 8%) were reintubated within the first 1 to 2 days whereas the other 4 cases (21.2%) were reintubated on the 4th, 5th, 6th, and 7th days post-extubation, respectively.

The association between post-extubation modes used and clinical and demographic characteristics is shown in Table 3. Eleven babies needed reintubation after being extubated to NCPAP (11.2%) and seven were reintubated after extubation to NIPPV (9.2%), none of those who were extubated to NC required reintubation (*P* = 0.203). Significant differences between the three modes involved the following parameters: air leak, need for PPV during resuscitation, surfactant administration, number of surfactant doses, PDA, HsPDA, BPD, birth weight, and gestational age (Table 3).

**Table 3:** Association between post-extubation mode and other parameters

Parameters	NCPAP ***N*** = 97	NIPPV ***N*** = 77	NC ***N*** = 26	***P***-Value
Birth Weight (gm)	1686.7 ± 500.8	1408.5 ± 494.9	2101.9 ± 505.5	< 0.0001
(mean ± SD)	1555 (1342.5,	1320 (1020,	2140 (1631.3,	
Median (IQR)	2011.3)	1685)	2412.5)	
Birth Weight (gm)				
> 1000 g	93 (95.9)	60 (77.9)	26 (100)	< 0.0001
≤ 1000 g	4 (4.1)	17 (22.1)	0 (0)	
Gestational Age (weeks)	31.2 ± 2.4	30 ± 2.3	33.6 ± 2.1	< 0.0001
Median (IQR)	31 (29, 33)	29 (28, 31)	34 (32, 36)	
Apgar Score at 1 min	6.7 ± 2.3	6.7 ± 1.9	6.4 ± 3.1	0.891
Median (IQR)	7 (5, 9)	7 (5, 8)	8 (4.5, 9)	
Apgar Score at 5 min	8.6 ± 1.6	8.5 ± 1.1	8.4 ± 1.9	0.934
Median (IQR)	9 (8, 10)	9 (8, 9)	9 (7.5, 10)	
Duration of Mechanical Ventilation (days)	2.4 ± 2.3	3.4 ± 4.1	2.7 ± 2.8	0.105
Median (IQR)	1 (1, 3)	2 (1, 4)	2 (1, 3.3)	
Duration of Non-Invasive Ventilation (days)	3.7 ± 5.3	3.6 ± 4.2	2.7 ± 2.4	
Median (IQR)	2 (1, 4)	2 (1, 4)	2 (1, 3.8)	0.786
Days till reintubation	1.9 ± 1.85	2.7 ± 1.9	–	0.446
Median (IQR)	1 (1, 2)	2 (1, 4)		
Age at Extubation (day)	2.52 ± 2.55	3.41 ± 3.91	3.56 ± 2.62	0.115
Median (IQR)	1 (1, 4)	2 (1, 4.3)	4 (1, 4.5)	
Maternal GBS Status				
Negative	89 (91.8)	67 (88.2)	25 (96.2)	0.331
Positive	6 (6.2)	9 (11.8)	1 (3.8)	
Unknown	2 (2.1)	0 (0)	0 (0)	
Maternal Antenatal Steroids				
No	46 (47.9)	35 (45.5)	16 (61.5)	0.356
Yes	50 (52.1)	42 (54.5)	10 (38.5)	
Need for PPV during resuscitation				
No	44 (45.8)	22 (28.9)	13 (50)	0.042
Yes	52 (54.2)	54 (71.1)	13 (50)	
Need for resuscitation medication.				
No	95 (97.9)	77 (100)	25 (96.2)	0.309
Yes	2 (2.1)	0 (0)	1 (3.8)	
Surfactant Administration				
No	21 (21.6)	9 (11.7)	20 (76.9)	< 0.0001
Yes	76 (78.4)	68 (88.3)	6 (23.1)	
Number of Surfactant Doses				
1	57 (75)	35 (52.2)	6 (85.7)	0.022
2	18 (23.7)	26 (38.8)	1 (14.3)	
3	1 (1.3)	6 (9)	0 (0)	
Need for intubation during resuscitation.				
No	52 (53.6)	40 (51.9)	14 (53.8)	0.972
Yes	45 (46.4)	37 (48.1)	12 (46.2)	
Mode of Delivery				
Vaginal delivery	19 (19.8)	8 (10.4)	7 (26.9)	0.095
Caesarian Section	77 (80.2)	69 (89.6)	19 (73.1)	
Pre-extubation Ventilation Mode				
PC-AC + VG	90 (96.8)	73 (96.1)	24 (96)	0.416
SIMV	3 (3.2)	1 (1.3)	1 (4)	
HFOV	0 (0)	2 (2.6)	0 (0)	
Patent Ductus Arteriosus (PDA)				
No	81 (83.5)	54 (70.1)	23 (88.5)	0.044
Yes	16 (16.5)	23 (29.9)	3 (11.5)	
HsPDA*				
No	11 (64.7)	3 (13.0)	2 (66.7)	0.009
Yes	6 (35.3)	20 (87.0)	1 (33.3)	
Reintubation				
No	87 (88.8)	71 (91.0)	26 (100)	0.203
Yes	11(11.2)	6 (9.0)	0 (0)	
Necrotizing Enterocolitis (NEC)				
No	92 (94.8)	69 (89.6)	25 (96.2)	0.322
Yes	5 (5.2)	8 (10.4)	1 (3.8)	
Intestinal Perforation				
No	96 (99)	74 (96.1)	26 (100)	0.300
Yes	1 (1)	3 (3.9)	0 (0)	
Air Leak				
No	88 (90.7)	72 (93.5)	19 (73.1)	0.011
Yes	9 (9.3)	5 (6.5)	7 (26.9)	
BPD				
No	93 (95.9)	65 (84.4)	26 (100)	0.006
Yes	4 (4.1)	12 (15.6)	0 (0)	
BPD Grade				
Mild	2 (50)	9 (75)		0.350
Moderate	2 (50)	3 (25)		
Sepsis status				
No	94 (96.9)	74 (96.1)	26 (100)	0.922
Yes	3 (3.1)	3 (3.9)	0 (0)	

Categorical data values are presented in n (%) and quantitative data values are presented in mean ± standard deviation (SD). Median and IQR were used for skewed or non-normal data.

Yates corrected Chi-square test was applied in case of small cell frequencies (50% or more cells have expected frequencies < 5), whereas the quantitative outcome measures were compared by using one-way analysis of variance (ANOVA) test or Kruskal–Wallis test (for skewed data) as appropriate to compute respectively statistical P-value

* HsPDA rate was computed among patients who had positive PDA (*n* = 48)

This is a retrospective study design and for some parameters, the data values were incomplete due to the unavailability of the information in the patients’ record files and thus all the percentages values computed using non-missing values

PDA was present only in 22.2% (48/216) of the neonates in our study. Of those 27 (56.3%) were having hemodynamically significant PDA (HsPDA) [14, 15]. HsPAD status observed was significantly higher in NIPPV 87% (20/23) compared to both NCPAP 35.3% (6/17) and NC 33.3% (1/3) (Yates corrected Chi-square *P* = 0.009) as shown in Table 3. Among patients with PDA, HsPAD status in reintubation 57.1% (4/7) and non-reintubation 56.1% (23/41) groups didn’t show any statistically significant differences (*P* = 0.959).

No statistically significant difference was observed in sepsis status between reintubation 5.6% (1/18) and non-reintubation 3.1% (6/192) groups (*P* = 0.583). Similarly, when compared sepsis status across three post-extubation groups showed insignificant difference (NCPAP 3.1%, vs NIPPV 3.8% vs NC 0% *P* = 0.922) (Table 3).

One-way ANOVA indicated that the mean age at extubation in days was not significantly different across three post-extubation modes (NCPAP 2.52 ± 2.55 vs NIPPV 3.41 ± 3.91 vs NC 3.56 ± 2.62; *P* = 0.115) (Table 3). Similarly, the mean age at extubation in days was observed to be slightly higher in those who were reintubated 4.17 ± 5.85 (median 1.5; IQR 1, 5) vs 3.19 ± 3.61 (median 2; IQR 1, 4), however, this difference was statistically insignificant (*P* = 0.495).

The reintubation rate was not affected by extubation to any form of non-invasive ventilation (OR = 0.78, 95% CI 0.26, 2.12, *P* = 0.625). Those 18 babies who required reintubation are 17 out of the 198 babies (8.6%) extubated from PC-AC + VG and 1 out of 4 (20%) extubated from SIMV. None of those who were extubated from High-Frequency Oscillatory Ventilation (HFOV) required reintubation. The mode of ventilation before extubation does not affect the reintubation rate (*P* = 0.877). Reintubation had multiple significantly different characteristics compared to the non-reintubation group including NEC, the method of delivery, BPD, and length of stay with a *P*-value ranging (from 0.02–0.003). NEC happened after reintubation in all cases. The presence of PDA and NEC was strongly associated with reintubation which increased by two and four-folds respectively in those morbidities (Table 4).

**Table 4:** Factors associated with Reintubation: Univariate logistic regression analysis

Parameters	Reintubation ***N*** = 18	Unadjusted Odds ratio (95% CI)	***P***-Value
Gestational Age (weeks)			
> 30 weeks	7 (5.6)	1.0	
≤ 30 weeks	11 (11.5)	2.16 (0.81, 5.81)	0.126
Apgar Score at 1 min	6.2 ± 2.4	0.92 (0.76, 1.12)	0.408
Median (IQR)	6 (4.5, 8)		
Apgar Score at 5 min	8.3 ± 1.8	0.91 (0.68, 1.21)	0.909
Median (IQR)	9 (7.8, 10)		
Length of hospital stay (days)	61.9 ± 59.2	1.02 (1.01, 1.03)	0.003
Median (IQR)	35 (23.5, 91)		
Duration of Mechanical Ventilation (days)	4.4 ± 6	1.11 (0.99, 1.24)	0.062
Median (IQR)	2 (1, 6)		
Duration of NCPAP (days)	3.1 ± 3	0.97 (0.85, 1.11)	0.673
Median (IQR)	2 (1,4)		
Duration of NIPPV (days)	7.8 ± 13.3	1.15 (0.98, 1.36)	0.088
Median (IQR)	3 (1.8, 11)		
Maternal Antenatal Steroids			
No	10 (9.3)	1.0	
Yes	8 (7.2)	0.76 (0.29, 2.01)	0.761
Need for PPV during resuscitation			
No	4 (4.3)	1.0	
Yes	14 (11.1)	2.75 (0.87, 8.65)	0.084
Surfactant Administration			
No	4 (6.1)	1.0	
Yes	14 (9.2)	1.6 (0.50, 4.97)	0.441
Number of Surfactant Doses			
1	9 (9.1)	1.0	
2	4 (8.7)	0.95 (0.28, 3.27)	0.938
3	1 (14.3)	1.67 (0.18, 15.43)	0.653
Need for intubation during resuscitation.			
No	9 (7.4)	1.0	
Yes	9 (9.2)	1.26 (0.48, 3.30)	0.641
Mode of Delivery			
Vaginal delivery	7 (18.4)	1.0	
Caesarian Section	11 (6.1)	0.29 (0.10, 0.80)	0.017
Pre-extubation Ventilation Mode			
PC-AC + VG	17 (8.6)	1.0	
SIMV or HFOV	1 (20)	1.18 (0.14, 9.91)	0.877
PDA			
No	11 (6.5)	1.0	
Yes	7 (14.6)	2.44 (0.89, 6.68)	0.083
Post-extubation mode			
NCPAP	11 (11.2)	1.0	
NIPPV	7 (9.0)	0.78 (0.29, 2.11)	0.625
Necrotizing Enterocolitis (NEC)			
No	13 (6.5)	1.0	
Yes	4 (23.5)	4.45 (1.27, 15.59)	0.020
Intestinal Perforation			
No	16 (7.5)	1.0	
Yes	1 (20)	3.06 (0.32, 29.05)	0.330
Air Leak			
No	14 (7.1)	1.0	
Yes	3 (13.6)	2.05 (0.54, 7.79)	0.291
BPD			
No	14 (7)	1.0	
Yes	4 (23.5)	4.11 (1.18, 14.28)	0.026
BPD Grade			
Mild	1 (9.1)	1.0	
Moderate	3 (50)	10.0 (0.74, 135.33)	0.083
Birth Weight (gm)			
> 1000 g	13 (6.6)	1.0	
≤ 1000 g	5 (22.7)	4.19 (1.32, 13.15)	0.009

*CI* Confidence interval

Outcome variable: the non-reintubation group was considered as the reference group

Categorical data values are presented in n (%) and quantitative data values are presented in mean ± standard deviation (SD)

Chi-square Fisher Exact test was used for 2 × 2 tables and tables more than 2 × 2, Yates corrected Chi-square test was applied in case of small cell frequencies (50% or more cells have expected frequencies < 5)

This is a retrospective study design and for some parameters, the data values were incomplete due to the unavailability of the information in the patients’ record files, and thus all the percentages values were computed using non-missing values

Using multivariate logistic regression, we found that the NEC (OR = 5.52, 95% CI 1.26, 24.11, *P* = 0.023) and the delivery by vaginal delivery (OR = 0.23, 95% CI 0.07, 0.78, *P* = 0.018) remained significantly associated with reintubation after controlling and adjusting for all other potential associated factors, confounders and predictors. The need for PPV during resuscitation was associated with reintubation but that association was statistically insignificant (OR = 4.60, 95% CI 0.97, 21.79, *P* = 0.055) (Table 5).

**Table 5:** Predictors associated with Reintubation: Multivariate logistic regression analysis

Predictors	Adjusted Odds ratio (OR)	95% CI for OR	P-value
**Necrotizing Enterocolitis (NEC)**			
No	1.0		
Yes	5.52	1.26, 24.11	0.023
**Need for PPV during resuscitation**			
No	1.0		
Yes	4.60	0.97, 21.79	0.055
**Mode of Delivery**			
Vaginal delivery	1.0		
Caesarian Section	0.23	0.07, 0.78	0.018

*CI* Confidence interval

Outcome variable: Non-reintubation was considered as the reference group

**Fig. 1:**
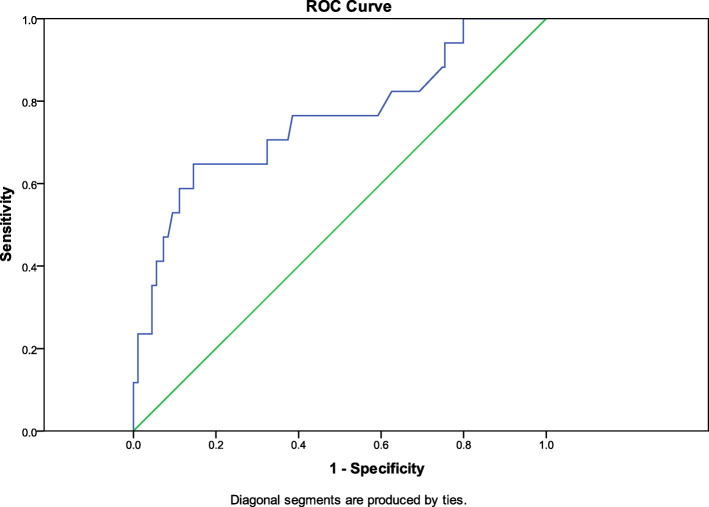
Receiver operating characteristic curve (ROC) to evaluate and assess the predictive accuracy of the developed logistic regression model (using the predicted probabilities). Multivariate logistic regression indicated that the final model demonstrated a modest fit (area under the curve (AUC) = 0.68, 95% CI 0.52, 0.82) (Fig. 1 ROC curve) and included the potential predictors and risk factors for reintubation as shown in Table 5

Finally, we computed a prediction model to evaluate the discriminative ability of potentially significant predictors that precisely predict reintubation. Multivariate logistic regression indicated that the final regression model demonstrated a modest fit (area under the curve (AUC) = 0.68, 95% CI 0.52, 0.82) (Fig. 1 ROC curve) and included the potential predictors and risk factors for reintubation.

## Discussion

The increasing use of non-invasive respiratory support strategies after birth as a part of the safe landing strategies is becoming the trend in NICUs across the world. Prolonged mechanical ventilation can be associated with significant morbidity in preterm neonates and the practice of prompt weaning and early extubation to non-invasive respiratory support has been the focus and ultimate goal since the past decade [4, 5, 11, 16].

Early extubation of the very low birth weight (VLBW) infant holds the potential for several benefits for the infant, including a decrease in calorie consumption, decreased tracheal and laryngeal injury, decreased nosocomial pneumonia, and sepsis, and a decreased incidence and severity of bronchopulmonary dysplasia. Non-invasive Ventilation in neonates has mainly been used to maintain effective breathing after a period of extubation and to avoid extubation failure. There has also been a recent trend to use NIPPV as the primary mode of ventilation for the early management of Respiratory Distress Syndrome (RDS) as an alternative to intubation and ventilation, but evidence of its superiority over traditional CPAP and ventilation is still lacking [17].

We conducted this study to compare the rate of reintubation after extubation to different non-invasive modes of ventilation.

Our study reported an 8.2% reintubation rate within 1 week after extubation. This is lower than the 19.6% reintubation rate after 48 h of extubation reported in a study by Chico et al. [18]. Necrotizing Enterocolitis was significantly associated with increased risk of reintubation, however, mode of delivery (cesarean section) was found to be significantly associated with decreased risk of reintubation in this study sample. A study from Brazil investigating extubation failure and reintubation reported age at extubation and low five-minute Apgar score to be associated with reintubation, while the length of mechanical ventilation, the potential of hydrogen (pH), and partial pressure of oxygen to be associated with extubation failure [19]. A prospective observational study conducted on 51 neonates, aimed to determine predictors for extubation failure, reported no significant difference between those that passed extubation (80%) and those that needed reintubation (20%) on all variables studied (clinical characteristics, maximum ventilator requirements, laboratory parameters), except for minute ventilation which was found to be significantly higher among those that failed extubation [19].

This study compared reintubation rates among preterm babies after extubation to either NCPAP or NIPPV. The findings showed no significant difference in reintubation rates between those extubated to NCPAP and those to NIPPV. This is in contrast to what four randomized controlled trials [20] using NIPPV have shown. These studies showed a significant decrease in extubation failures as compared with NCPAP. The exact mechanisms by which NIPPV improves efficacy are not known.

Our study also investigated the rates of gastrointestinal perforation, NEC, and air leak in both groups. There was also no difference concerning gastrointestinal perforation and NEC when comparing NIPPV, NCPAP, and NC. From our results, NEC is associated with reintubation, but it is not a complication of one mode of ventilation. Pneumothorax was significantly higher in those who were extubated to NC compared to those extubated to NCPAP and NIPPV. It might be related to the extra effort done by babies extubated to NC to maintain their Functional Residual Capacity (FRC).

A meta-analysis that covered three clinical trials reported a statistically significant benefit for those extubated to NIPPV compared to NCPAP, regarding preventing extubation failure, along with the absence of gastrointestinal perforation [11]. A systematic review of a larger number of studies showed a decrease in extubation failure along with the need for re-intubation (48 h-7 days) with NIPPV when compared to NCPAP [9, 21, 22]. Another meta-analysis reported that NIPPV is more efficient when compared to NCPAP regarding respiratory failure or as related to the need for reintubation [8, 10].

The association between extubation failure in preterm babies and the type of delivery was not clearly understood. Many researchers did not find any association between extubation failure and the type of delivery [23–26]. Teixeira et al. found a similar association to ours between vaginal delivery and reintubation in preterm delivery [27]. The exact mechanism is not well known; however, it might be related to the preterm labor without the needed requisite care, thereby predisposing the newborn to a worse clinical condition. Besides, the vaginal delivery itself occurs in most cases due to spontaneous preterm labor which is due to an underlying inflammatory or infectious condition [27–29].

The findings of this study can be attributed to a range of predictors and covariates including the nature of the current study, being retrospective which is the main limitation. Despite not, all have been reintubated, the relatively high number of intubated babies in the gestational age group of infants included in the study reflects the tendency of our NICU team to intubate those infants. Less Invasive Surfactant Administration (LISA) has been introduced recently to our clinical practice. With that, we expect a significant decrease in the number of intubated babies and subsequently the number of extubation failures [30–32]. Besides, the gestational age group of infants included in the study (≥ 28 weeks gestation) might be a contributing factor for not finding a statistically significant difference between reintubation rates in NCPAP versus the NIPPV group. A proper case-control study or randomized controlled trial with a larger sample size including smaller gestational ages is needed to deeply investigate the reintubation rates among those extubated to NCPAP versus NIPPV and to confirm other potential predictors associated with reintubation.

## Conclusion

Reintubation rates were less with NIPPV when compared with NCPAP, however, this difference was not statistically significant. This study highlights the need for further research studies with a larger number of neonates in different gestational ages birth weight categories. Ascertaining this information will provide valuable data for the factors that contribute to re-intubation rates and influence the decision-making and management of RDS patients in the future.

## Data Availability

The datasets used and/or analyzed during the current study are available from the corresponding author on reasonable request.

## Abbreviations

RDS: Respiratory Distress Syndrome
NCPAP: Nasal Continuous Positive Airway Pressure
NIPPV: Nasal Intermittent Positive Pressure Ventilation
NC: Nasal Cannula
NEC: Necrotizing Enterocolitis
BPD: Bronchopulmonary Dysplasia
DM: Diabetes Mellitus
GBS: Group B Streptococcus
PROM: Premature Rupture of Membranes
FRC: Functional Residual Capacity
PPV: Positive Pressure Ventilation
HFOV: High-Frequency Oscillatory Ventilation
SIMV: Synchronized Intermittent Mandatory Ventilation
PDA: Patent Ductus Arteriosus
LISA: Less Invasive Surfactant Administration
FiO2: Fraction of Inspired Oxygen
pH: potential of Hydrogen
PTB: Preterm Birth
VD: Vaginal Delivery
CS: Cesarean Section
GA: Gestational Age
BW: Birth Weight
AUC: Area Under the Curve
OR: Odds Ratio
CI: Confidence Interval
WWRC: Women’s Wellness and Research Center
HMC: Hamad Medical Corporation
NICU: Neonatal Intensive Care Unit
MRC: Medical Research Center
